# Etymologia: *Falciparum*

**DOI:** 10.3201/eid2702.ET2702

**Published:** 2021-02

**Authors:** Aparna Tiwari, Abhinav Sinha

**Affiliations:** Council of Scientific and Industrial Research, New Delhi, India (A. Tiwari);; India Council of Medical Research–National Institute of Malaria Research, New Delhi (A. Tiwari, A. Sinha)

**Keywords:** etymologia, Falciparum, species, Plasmodium, parasites, malaria, humans

## *Falciparum* [fal-′sɪ-pə-rəm]

From the Latin *falx* or *falci* (sickle or scythe-shaped) and *parum* (like or equal to another) or *parere* (to bring forth or bear). The species *falciparum* in the genus *Plasmodium* is the parasite that causes malignant tertian malaria in humans (Figure).

**Figure:**
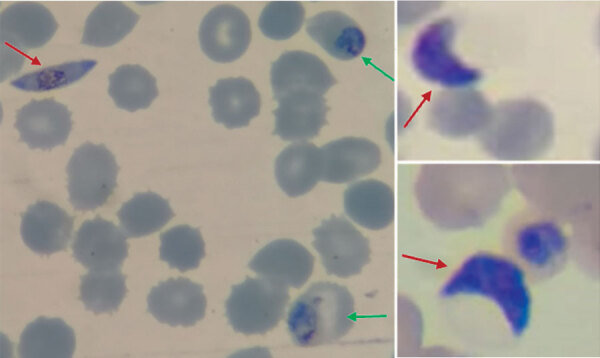
Giemsa-stained thin blood smear of *Plasmodium falciparum* (original magnification ×1,000). Red arrows indicate gametocytes, and green arrows indicate trophozoite stages. Photograph provided by A. Tiwari.

There were many terms suggested for this parasite, such as *Ematozoo falciforme* by Antolisei and Angelini in 1890 and *Haemotozoon falciforme* by Thayer and Hewetson in 1895, because of its sickle-shaped gametocytes, the sexual stage of *falciparum* parasites. However, the term *falciparum*, suggested by William Henry Welch in 1897, was eventually accepted. In 1954, *Plasmodium falciparum* (previously *Laverania malariae*) was approved by International Commission on Zoological Nomenclature.
